# The use of iloprost in the treatment of ‘out of proportion’ pulmonary hypertension in chronic obstructive pulmonary disease

**DOI:** 10.1007/s11096-013-9762-3

**Published:** 2013-04-03

**Authors:** Bartosz Lasota, Szymon Skoczyński, Katarzyna Mizia-Stec, Władysław Pierzchała

**Affiliations:** 1 2nd Department of Cardiology, Medical University of Silesia, Katowice ul. Ziołowa 45/47, 40-635 Katowice, Poland; 2 Department of Pulmonology, Medical University of Silesia, Katowice ul. Medyków 14, 40-752 Katowice, Poland; 3 1st Department of Cardiology, Medical University of Silesia, Katowice ul. Ziołowa 45/47, 40-635 Katowice, Poland

**Keywords:** Chronic obstructive pulmonary disease, Heart failure, Iloprost, Prostanoids, Pulmonary hypertension

## Abstract

*Case* Pulmonary hypertension secondary to respiratory disease most often occurs as a complication of chronic obstructive pulmonary disease, which currently constitutes one of the leading causes of death. Some patients with hypoxaemia reveal “out of proportion” pulmonary hypertension with inappropriate increase of pulmonary artery pressure. Iloprost, analogue of prostacyclin, dilates systemic vessels and pulmonary vessels in particular if administered by inhalation. It appears to be important, life-saving, complementary therapy. However, there is no evidence for its routine use in out of proportion arterial pulmonary hypertension. This case study presents a 44-year old man with chronic obstructive pulmonary disease and “out of proportion” pulmonary hypertension. We present the results of his treatment with iloprost. *Conclusion* In a patient with “out of proportion” pulmonary hypertension due to chronic obstructive pulmonary disease, inhaled iloprost led to improvement in clinical status and echocardiographic parameters, including a reduction of right ventricular systolic pressure.

## Impact of findings on practice

- Inhaled iloprost in ‘out of proportion’ pulmonary hypertension due to chronic obstructive pulmonary disease may reduce symptoms as well as improve echocardiographic parameters.
- Inhaled iloprost seems to be safe in ‘out of proportion’ pulmonary hypertension due to hypoxaemia.

## Introduction

Pulmonary hypertension (PH) is a haemodynamic and pathophysiologic state characterized by a mean pulmonary artery pressure (mPAP) above 25 mmHg when measured during right heart catheterization (RHC). PH has no pathognomonic signs or symptoms being therefore difficult to diagnose. Furthermore, determining if PH is secondary to heart failure (HF) or respiratory disease is not always straightforward.

PH secondary to respiratory disease occurs most frequently as a complication of chronic obstructive pulmonary disease (COPD). Mild or moderate PH is common in COPD, but only a small percentage of patients with COPD develop severe or so called “out of proportion” PH defined as a mPAP above 40 mmHg [1].

Iloprost is a prostacyclin PGI2 analogue for oral, intravenous or inhaled administration. It causes dilatation of pulmonary and systemic arteries. The inhalation route seems to be more selective effect over the pulmonary circulation. There is no evidence for routine use of vasodilatators, such as iloprost, in cases of “out of proportion” PH with hypoxaemia.

## Case history

A 44-year-old male with symptoms of circulatory and respiratory failure, complained of gradual deterioration in exercise tolerance, increased shortness of breath and legs oedema for several weeks was admitted to the Department of Cardiology of the Medical University of Silesia in Katowice, Poland. He had had a diagnosis of HF, but was taking no treatment for it. He smoked (60 pack-years) and consumed alcohol. On admission he had severe dyspnoea (NYHA/WHO class IV). Physical examination revealed central and peripheral cyanosis, systolic murmur all over precordial area with punctum maximum in the crossing of fourth intercostal space and right sternal line, decreased breath sounds with widespread wheezing with superimposed fine bilateral basal crackles. Blood pressure was elevated—BP 160/80 mmHg. In the laboratory studies, the basic abnormalities were as follows: increased NT-proBNP (See Table 1), polycythaemia (haemoglobin 18.6 g/dl), thrombocytopaenia (platelets 108.000/mm^3^) and slight increase in the markers of myocardial damage (troponin T 0.014 ng/ml, creatine kinase-MB 42 U/l). C-reactive protein and D-dimer were within normal range. The arterial blood gas examination confirmed acute on chronic respiratory failure: pH 7.31, pO_2_ 41 mmHg, pCO_2_ 77 mmHg, HCO_3_ 37 mmol/l, SaO_2_ 79 %.

**Table 1:** Echocardiographic and laboratory parameters associated with pulmonary hypertension during iloprost treatment

Parameter	Baseline	Follow-up
*Echocardiographic*		
Right ventricular outflow tract [mm]	52	39
Right ventricular inflow tract [mm]	62	41
Trigeminal ring [mm]	53	31
Tricuspid annular plane systolic excursion [mm]	14	18
Right atrium [cm^2^]	30	21
Inferior vena cava [mm]	29	18
Respiratory collapsability of inferior vena cava [%]	<50	>50
Pulmonary trunk [mm]	30	25
Systolic pulmonary artery pressure [mmHg]	70	36
*Laboratory*		
NT-proBNP [pg/ml]	2938	921

ECG revealed sinus rhythm, with a pulse of 62/min, dextrogram, P pulmonale and right bundle branch block. Echocardiography showed dilatation of the right ventricule and right atrium (See Table). Right ventricular function was depressed and there was a decreased value of tricuspid annular plane systolic excursion (TAPSE). The pulmonary trunk was extended. The left ventricular diameters were within normal range: end-diastolic diameter −45 mm, end-systolic diameter −19 mm. Ejection fraction was preserved (LVEF 55 %). Paradoxical interventricular septal motion was present. Mitral inflow suggested mild form of diastolic left ventricle dysfunction with E/E’ ratio = 11. Interatrial septum was tinned and displaced towards the left atrium. These abnormalities were accompanied by severe trigeminal regurgitation, dilatation of trigeminal ring and additional features of PH: increased systolic pulmonary artery pressure and moderate pulmonary valve regurgitation (short acceleration time −90 ms). The hepatic veins were extended with reversed flow and the inferior vena cava diameter increased with a small tidal variability. The chest X-ray image showed increased lung markings and enlarged cardiac silhouette. The angio-CT confirmed enlarged cardiac silhouette characteristic of right ventricular dysfunction. There was no evidence of pulmonary embolism. Spirometry showed severe obstruction: forced expiratory volume in 1 s FEV1[l] = 1.57; FEV1[%] = 41, per = 1; forced vital capacity FVC[l] = 3.26; FVC[%] = 70, per = 1; FEV1 %FVC = 0.48, per = 1. The treatment protocol consisted of diuretics (spironolactone 1 × 100 mg, furosemide 3 × 40 mg), acetylsalicylic acid (1 × 75 mg), ramipril (1 × 5 mg), carvedilol (2 × 3.125 mg), nadroparin (1 × 7600 IU AXa), bronchodilatators: formoterole 2 × 12 μg, tiotropium 1 × 18 μg, theophylline 1 × 300 mg and oxygen therapy through nasal prongs adjusted according to the blood gases. As there was no improvement with standard treatment, RHC was performed, showing the following mean pressures: right atrial pressure (RAP) 13 mmHg, right ventricular pressure (RVP) 13 mmHg, mPAP 40 mmHg, pulmonary capillary wedge pressure (PCWP) 21 mmHg. On the basis of RHC, the diagnosis of PH was set. Given the very serious condition and young age of the patient it was decided to start off label on the 3rd day, together with conventional treatment, nebulized iloprost (6 × 5 μg), which resulted in clinical, echocardiography and laboratory improvement [2]. There was a gradual improvement from the 2nd day of usage. The parameters on the 3rd day of administration were as follows: the echocardiography revealed regression of right ventricular dilatation and diameter of pulmonary artery (See Table). The echocardiography doppler showed mild trigeminal valve regurgitation with a decreased ring diameter and TAPSE, trace pulmonary valve regurgitation, decreased inferior vena cava diameter with an intact respiratory variation. The NT-proBNP decreased significantly and the patient was finally able to perform 6MWT. He reached the distance of 320 m, without significant desaturation (SaO_2_ min = 91 %) achieving 10/20 points in Borg scale at the end of the test. There were no observed complications of iloprost treatment (no drop in arterial blood pressures, no further worsening of respiratory failure). After a significant improvement in the general condition, iloprost therapy was discontinued on the 4th day and the patient was referred to the Department of Pulmonology in order to continue pulmonary diagnosis the respiratory workup, and further management.

## Discussion

The presented case history is a unique example of successful iloprost therapy in acute cardio-respiratory failure in a patient with “out of proportion” PH due to COPD. Only in a small percentage of patients (<5 %) PH is disproportionately high called “out of proportion” [1]. This group of patients may probably obtain additional benefits during specific treatment for pulmonary artery hypertension (PAH). The primary target for PH treatment in a background of COPD is to ensure normalization of arterial blood gases with the use of bronchodilators, oxygen or noninvasive positive pressure therapy if needed. As seen in our patient’s case history: clinical symptoms, parameters of right ventricular overload and dysfunction, right ventricular systolic pressure (RVSP) were not improved with conventional therapy. The role of specific drugs is justified on a pathophysiological basis, by their ability to improve the impaired endothelial balance. Calcium channel blockers have a potential role in decrease of mPAP, but they are often inadequate in patients with COPD, because of worsening of hypoxaemia. Similar adverse effects were observed after receptor antagonists for the endothelin-1, sildenafil and prostanoids were administered iv [1]. Deterioration of gas exchange occurs due to reduction of hypoxaemic vasoconstriction in the pulmonary vasculature followed by worsening of the ventilation to perfusion mismatch. In addition, these are drugs that do not act selectively over the pulmonary circulation. Different effects were observed with inhaled therapy with prostanoids, such as iloprost, used in our patient. Iloprost, approved for the treatment of patients with PAH, is a potent vasodilatator with a run time of approximately 60 min. It plays an important role in PH treatment due to its antiproliferative and antithrombotic action. In COPD following exposure to tobacco, where there is endothelial dysfunction and impaired vasodilatation, inflammation, proliferation and apoptosis, iloprost during in vitro studies showed its protective effect on pulmonary vasculature. Iloprost preferentially dilates the pulmonary circulation in areas that are well ventilated, improves ventilation perfusion matching and gas exchange [1, 3]. The inhaled drug reaches the best-ventilated lung areas which receive the highest dose [3]. Iloprost therapy did not lead to a significant decrease in spirometry parameters (FEV1, FVC, Total Lung Capacity), but improved 6MWT results [4]. In addition, iloprost provides better exercise tolerance in these patients, as confirmed by clinical data available [4, 5]. Inhaled administration avoids systemic adverse effects and serious haemodynamic consequences [3, 4]. Benefits of iloprost are particularly evident in periods of increased respiratory demand [5]. Another advantage is the simple apparatus for inhalation, no need for logistical supply, which undoubtedly improves patient comfort compared to subcutaneous treprostinil.

## Conclusion

We believe that our case story provides an impact on clinical practice and indicates iloprost as a good treatment option in severe clinical deterioration of PH. In our case, inhaled iloprost significantly improved the general condition of the patient, reduced symptoms as well as improving echocardiographic parameters, including the reduction of RVSP. Iloprost was safe in our case of “out of proportion” PH in a patient with respiratory failure due to COPD. This indicates an urgent need to perform a randomized clinical trial to test whether iloprost should be used as a recommended treatment in severe cases of “out of proportion” PH in patients with respiratory failure.
